# The clinical value of passive leg raising plus ultrasound to predict fluid responsiveness in children after cardiac surgery

**DOI:** 10.1186/s12887-021-02703-2

**Published:** 2021-05-19

**Authors:** Deqiang Luo, Wei Dai, Lei Lei, Xueying Cai

**Affiliations:** 1 Department of Intensive Care Unit, The First Affiliated Hospital of Nanchang University, No. 17 Yongwaizheng Street,Dong Lake District, 330000 Nanchang City, Jiangxi Province China; 2 Department of Intensive Care Unit, The Fifth People’s Hospital of Shangrao City, No.1, Jiannan Road, 334000 Shangrao City, Jiangxi province China; 3 Department of Animal Science, Hubei Vocational College Of Bio-Technology, No.1, Yezihu Lake, Hongshan District, 430070 Wuhan City, Hubei province China; 4 Department of Intensive Care Unit, Affiliated Hangzhou First People’s Hospital, Zhejiang University School of Medicine, No. 261 Huansha Road, Shangcheng District, 310006 Hangzhou City, Zhejiang Province China

**Keywords:** congenital heart surgery, fluid responsiveness, passive leg raising, ultrasound

## Abstract

**Background:**

There are few non-invasive monitoring methods that can reliably predict fluid responsiveness (FR) in children. Here, we interrogate the value of doppler ultrasound evaluation of passive leg raising (PLR)-induced changes in stroke volume (SV) and cardiac output (CO) as a predictor of FR in children with mechanical ventilation after congenital cardiac surgery.

**Methods:**

A total of 40 children with mechanical ventilation following congenital cardiac surgery, who required volume expansion (VE) were included in this study. Hemodynamic parameters such as heart rate (HR), mean arterial pressure (MAP), SV, and central venous pressure (CVP) were monitored before and after PLR and VE. Besides, we assessed changes in SV and CO by bedside ultrasound. Patients showing > 10 % increase in SV in response to VE were considered to be responders (26 patients), while the rest (14 patients) were defined as non-responders.

**Results:**

Our data demonstrated that ΔSV-PLR and ΔCO- PLR were positively correlated with ΔSV-VE (r = 0.683, *p* < 0.001 and r = 0.374, *p* = 0.017, respectively), and the area under the ROC curve (AUC) of ΔSV-PLR was 0.879 (95 % CI [0.745 1.000], *p* < 0.001). The best cut-off value for ΔSV-PLR in predicting FR was 13 %, with its sensitivity and specificity were 81.8 and 86.3 %, respectively. ΔCVP, ΔHR, and ΔMAP were weak predictors of FR in the children.

**Conclusions:**

Our study demonstrated that SV changes, as evaluated by noninvasive ultrasound combined with PLR, could effectively evaluate FR in children under mechanical ventilation after congenital cardiac surgery.

## Background

Proper fluid loading is pivotal in maintaining hemodynamic stability in children who undergo congenital cardiac surgery [1]. Classical fluid challenge methods that evaluate the FR create an extra fluid burden on patients with congenital heart disease which might result into serious side effects such as exacerbated tissue edema, organ failure, or mortality [2, 3]. Whereas Pulse index Continuous Cardiac Output (PiCCO), which integrates a wide array of both static and dynamic hemodynamic data such as SV, is considered to be a “gold standard”, it is expensive, invasive, and could cause catheter infection [4, 5]. Until now, there are few non-invasive monitoring methods that can reliably predict FR in children, except for respiratory changes in peak aortic flow velocity [6, 7]. It is, therefore, essential to explore a simple, effective, and non-invasive method for evaluating the FR and volume status in children.

Passive leg raising (PLR) is a simple repeatable self-replenishing fluid method done to shift venous blood from the lower limbs toward the intrathoracic compartment [8, 9]. Transthoracic Doppler echocardiography (TTE ) is a non-invasive method that allows for real-time monitoring of the descending aortic blood flow, and the estimation of SV [10, 11]. Therefore, using the bedside ultrasound to measure the SV variation triggered by PLR may be important in the evaluation of FR in children.

Here, we tested whether PLR related changes in SV and CO as monitored by bedside echocardiography could accurately predict FR in children with mechanical ventilation after cardiac surgery.

## Methods

### Ethical approval

The study was conducted in accordance with the Declaration of Helsinki and was approved by the Ethics Committee of Shangrao Fifth People's Hospital (No.2016-12-01). All participants gave written informed consent prior to their enrollment in this study.

### General information

This prospective observational study was carried out at the integrated Intensive Care Unit of the Fifth People’s Hospital of Shangrao City (Shangrao, China) from December 2016 to July 2017. We enrolled a total of 48 patients who were under mechanical ventilation following cardiac surgery, and whose attending physician conducted a fluid challenge. The execution of the fluid challenge dependent on at least one clinical sign of inadequate tissue perfusion, with no contraindications for infusion. The clinical signs of inadequate tissue perfusion were characterized by acute circulatory failure (30 % decrease in Mean arterial pressure [MAP], or need vasopressor drugs to maintain normal systolic blood pressure; 10 % increase in heart rate[HR] without arrhythmia; < 0.5 ml·kg^− 1^·h^− 1^ urine output for at least 1 h), mottled skin, oliguria (less than 0.5 ml·kg^− 1^·h^− 1^ diuresis), 1.5-fold increase in arterial blood lactic acid, acute kidney failure, or clinical and laboratory signs of extracellular dehydration [4, 12]. We excluded patients with clinical signs of hemorrhage, arrhythmia, PLR contraindication, less than 0.30 left ventricular ejection fraction, more than 40 mmHg pulmonary artery systolic pressure, or known allergic reaction to albumin.

### Echocardiography and Hemodynamic Data collection

A 4-5.5fr central venous catheter was inserted into the right internal jugular vein or the right/left subclavian vein to monitor CVP, while a 7 cm 3fr arterial catheter was inserted into the right or left femoral artery to monitor dynamic blood pressure, before operation. MAP was calculated as MAP = (systolic arterial pressure + 2diastolic artery pressure)/3. The standard transthoracic probe (3SP-D) of GE VIVIVIXE9 Doppler echocardiography was used to measure SV. On the five chambers apical view, the aortic blood flow was recorded using pulsed Doppler, with the sample volume placed on the annulus aorta. A velocity-time integral (VTI) of the aortic blood flow was also measured. The aortic valve area was calculated from the diameter of the aortic orifice, measured at insertion of the aortic cusps, as aortic area = π* (aortic diameter/2)^2^. SV and CO were measured with the equations: SV = VTI × aortic area, and CO = SV × HR. The aortic area was considered to be stable during the trial and was measured only once in the initial measurement. Every VTI measurement was taken based on two or three measurements in one breathing cycle. All of the measurements were conducted by a cardiologist.

### Study Design

The patients were placed in a supine position, with the upper body parts being 45° higher (Base 1) and four hemodynamic parameters (HR, blood pressure, SV, and CVP) were measured. Afterwards, the upper parts of the body were lowered to achieve a horizontal position and the lower extremities raised to 45° (Base 2), and then the four hemodynamic parameters were measured again within 30 s to 1 min. The patients were placed back to the initial position (the upper parts being 45° higher, Base 3) for 10 min and the hemodynamic parameters were remeasured. Besides, the four hemodynamic parameters were again measured immediately following the administration of the bolus of the intravenous fluid challenge using 10 ml/kg of 5 % albumin in 15 min. The patients showing > 10 % increase in SV in response to the VE were considered responders, while the rest were defined as non-responders. All the procedures were conducted while the patients were kept fully sedated, and the parameters such as the use of vasoactive drugs, sedatives and mechanical ventilation remained unchanged.

### Statistical analysis

Continuous variables were presented as the mean ± standard deviation if normally distributed, or as a median (range) if the distribution was not normal. Student’s unpaired t-test or the Mann–Whitney U-test was used to evaluate group differences. To assess the correlation between ΔSV-VE and PLR related ΔSV, ΔMAP, ΔHR, ΔCVP, or ΔCO, linear regression analysis was performed. Linear correlations were tested using the Pearson test and linear regression method. To determine the ability of all the variables to predict fluid responsiveness, receiver operating characteristic (ROC) curves were generated, and the area under the ROC curve was calculated. The ROC curves were compared using the Hanley–McNeil test [13]. Youden’s index was calculated as: Youden’s index = sensitivity + specificity – 1 [14]. A *p* < 0.05 was considered to be statistically significant. All the statistical analyses were performed with IBM SPSS 23.0 (SPSS, Inc, Armonk, NY).

## Results

### Patient Characteristics

A total of 48 patients who were under mechanical ventilation with presumed hypovolemia and were considered for VE were included in the study from December 2016 to December 2017. Among these eligible patients, 8 were excluded because of poor transthoracic insonation. Therefore, 40 patients (18 females and 22 males) with mean age of 5.41 ± 3.25 years were included in the study. There were patients with ventricular septum defect (VSD) (*n* = 18, 45 %), tetralogy of fallot (TOF) (*n* = 4, 10 %), atrial septal defect (ASD) (*n* = 11, 27.5 %), or ASD + VSD (*n* = 7, 17.5 %). There was no significant difference in age, gender, vasoactive drug usage score, ventilator parameters, and indication for ICU stay (*p* > 0.05). The clinical characteristics for the two groups are shown in Table 1.

**Table 1:** Patient characteristics. Values are expressed in frequencies (%), median (with IQR) or mean (± SD)

Characteristics	Responders (*n* = 26)	Non-responders (*n* = 14)	*p* value
Age(years)	3.76 ± 1.57	3.65 ± 1.54	0.36
Weight(kg)	8.50 ± 6.47	8.30 ± 7.50	0.91
Sex ratio n (%)			
Male	12(45.0)	6(45.0)	0.82
Female	14(45.0)	8(45.0)	
Sedation and analgesics use/not use	26/3	14/2	0.80
Ventilation			
Tidal volume (mL/kg)	7.50 ± 0.51	7.64 ± 0.63	0.44
PEEP(cmH_2_O)	5.19 ± 2.02	4.64 ± 1.21	0.55
Plateau pressure (cmH_2_O)	20.1 ± 2.0	20.3 ± 3.3	0.84
VIS	5.69 ± 2.42	4.07 ± 2.67	0.06
Cardiac function(GEF, %)	34 ± 4.5	32 ± 6.0	0.24
Indication for ICU stay n (%)			
VSD	12(46.2)	6(42.9)	0.84
ASD	8(30.8)	3(21.4)	0.53
VSD + ASD	3(11.5)	4(28.6)	0.18
TOF	3(11.5)	1(7.1)	0.66

*VSD* Ventricular septum defect, *ASD* atrial septal defect, *TOF* Tetralogy of Fallot, *VIS* vasoactive-inotropic score; *PEEP* positive end expiratory pressure; *GEF* Global ejection fraction

### Hemodynamic changes to PLR and VE

**Fig. 1:**
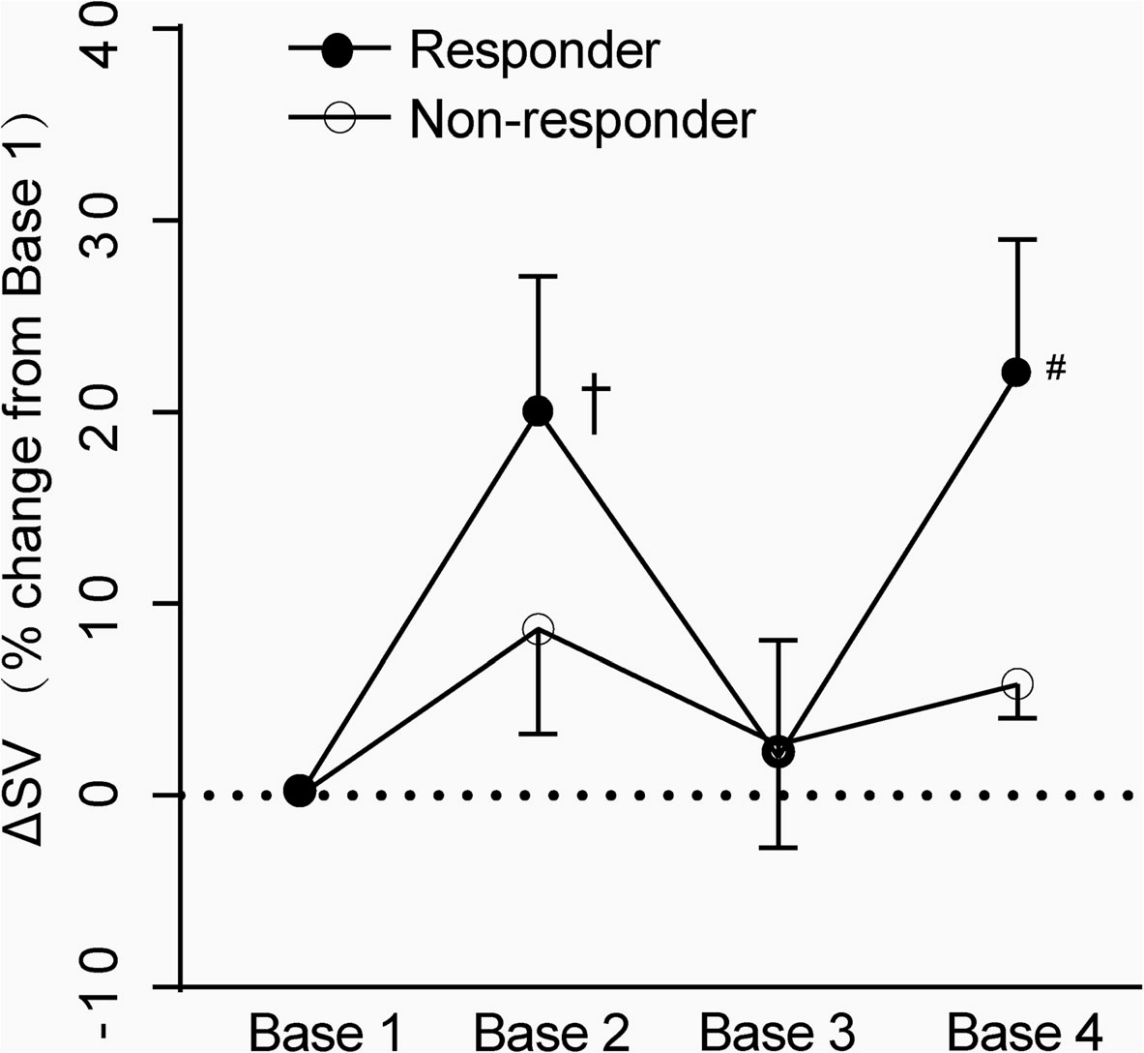
Evaluation of the ΔSV for the four steps of the study expressed as percentage change from Base 1. ^†^*p* < 0.05 vs.Base 1; ^#^*p* < 0.05 vs. Base 3

ΔSV in 26 children (responders) increased by more than 10 % from the base 3 value after VE, while ΔSV in 14 patients (non-responders) increased by less than 10 %. There were no significant shifts in all hemodynamic changes (*p* > 0.05) between Base 3 and Base 1. The hemodynamic parameters before and after PLR and VE are listed in Table 2; Fig. 1.

**Table 2:** Hemodynamic parameters in responders and non-responders

	Base 1	Base 2	^a^*p*	Base 3	^b^*p*	Base 4	^c^*p*
Reponders							
HR,Beats/min	133.1±11.3	128.5±12.2	0.16	132.2±10.8	0.77	128.3±11.2	0.21
MAP, mmHg	58.3±3.6	60.4±3.2	0.03	59.0±3.9	0.13	62.5±2.8	0.01
CVP, mmHg	9.3±0.7	10.7±1.3	0.00	9.6 ± 0.8	0.08	10.8 ± 1.2	0.00
CO, L/min	2.8±0.2	3.1±0.5	0.01	2.8 ± 0.3	1.00	3.2 ± 0.5	0.00
SV, mL	19.7±3.3	24.1±5.0	0.00	20.2±4.0	0.63	24.5±4.6	0.00
ΔSV (%)		20.1±7.0				22.1±7.0	
Non-responders							
HR,Beats/min	129.0±11.0	127.2±7.2	0.61	129.8±11.6	0.85	127.4±10.5	0.57
MAP, mmHg	61.3±3.6	65.6±4.9	0.49	61.7±3.8	0.78	66.0±4.2	0.01
CVP, mmHg	9.4±0.9	10.8±0.9	0.00	9.5 ± 1.4	0.82	10.4 ± 1.4	0.10
CO, L/min	2.8±0.3	3.0±0.6	0.27	2.8 ± 0.3	1.00	3.0 ± 0.4	0.15
SV, mL	22.1±3.8	23.6±3.6	0.29	22.8±4.7	0.67	24.1±5.0	0.48
ΔSV (%)		8.7±6.5				5.8±2.8	

*h* heart rate; *MAP* mean arterial pressure; *CVP* central venous pressure; *SV* stroke volume; *CO* Cardiac Output. ^a^*p* = Base 2 vs. Base 1; ^b^*p* = Base 3 vs. Base 1, ^c^*p* = Base 4 vs. Base 3; Values given as mean **±** SD

### Correlations and ROC curves

**Fig. 2:**
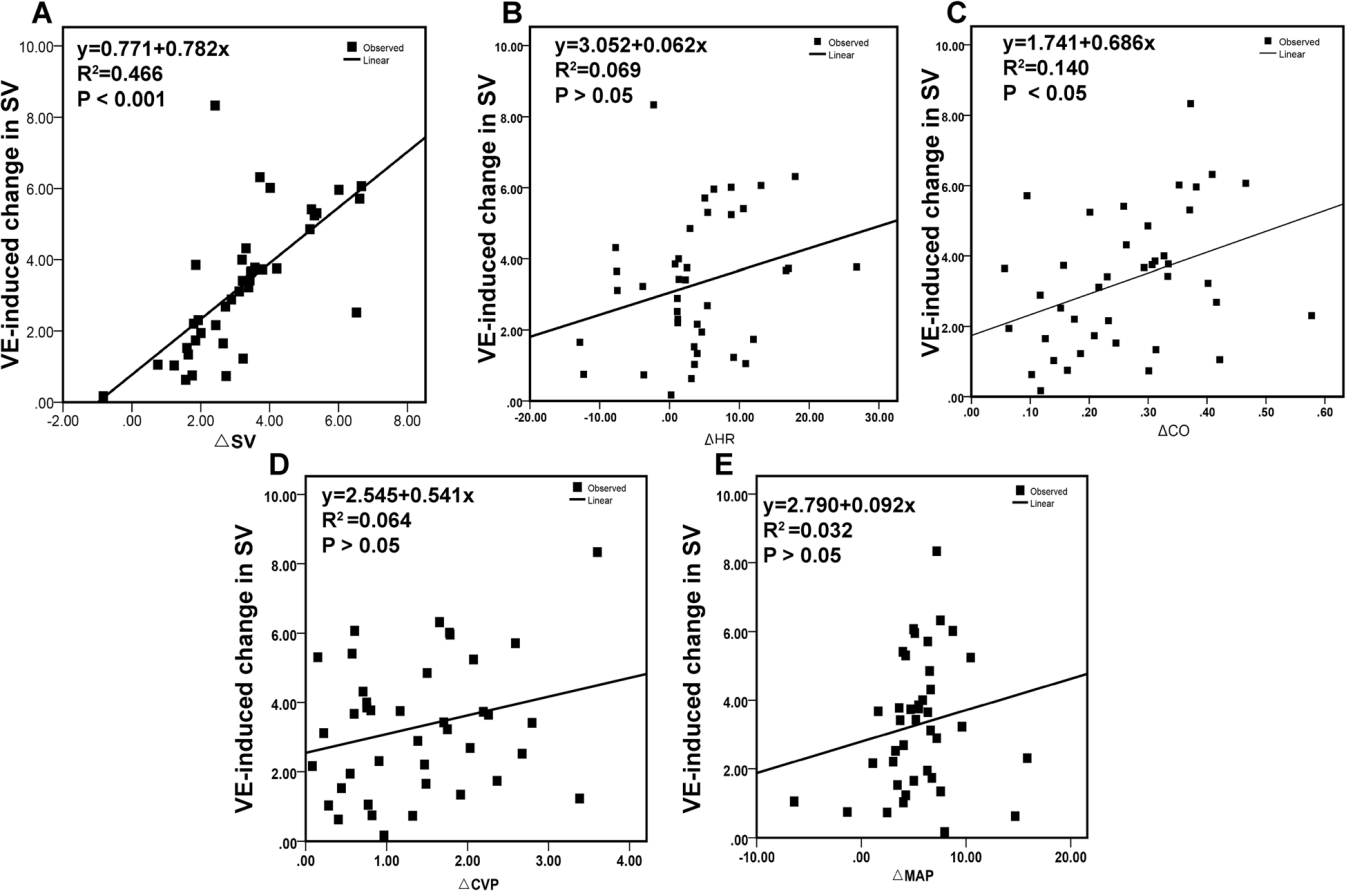
Linear correlation for all the parameters against ΔSV-VE **a** Correlation between ΔSV-PLR and ΔSV-VE. **b** Correlation between ΔHR-PLR and ΔSV-VE. **c** Correlation between ΔCO-PLR and ΔSV-VE. **d** Correlation between ΔCVP-PLR and ΔSV-VE. **e** Correlation between ΔMAP-PLR and ΔSV-VE. PLR: Passive leg raising; VE: Volume expansion

The ΔSV-PLR and ΔCO- PLR were positively correlated with ΔSV-VE (Pearson’s *r* = 0.683, *p* < 0.001 and r = 0.374, *p* = 0.017, respectively). The highest area under the ROC curve (AUC) for ΔSV-PLR (0.879 ± 0.069) successfully predicted FR in the patients (95 % CI [0.745 1.000], *p* < 0.001). The correlation between Δ-PLR and ΔSV-VE is shown in Table 3; Fig. 2.

**Table 3:** Diagnostic accuracy of index changes induced by PLR for predicting fluid responsiveness

Items	r	*p*	AUC	*p*	Threshold(%)	Sensitivity(%)	Specificity(%)	PPV(%)	NPV(%)
Δ SV	0.683	0.000	0.879	0.000	13	81	86	85	82
Δ CO	0.374	0.017	0.753	0.009	8	81	71	79	81
Δ HR	0.101	0.263	0.585	0.379	-5	46	78	68	59
ΔMAP	0.178	0.273	0.640	0.148	5	50	71	63	59
ΔCVP	0.253	0.116	0.530	0.755	18	39	71	57	54

*PLR* Passive leg raising; *ΔSV* PLRinduced change in stroke volume; *ΔCO* PLRinduced change in cardiac output; *ΔHR* PLRinduced change in heart rate; *ΔMAP* PLRinduced change in mean arterial pressure; *ΔCO* PLRinduced change in pulse pressure; *PPV* Positive predictive value; *NPV* Negative predictive value; *r* Correlation coeffcient between Δ-PLRand ΔSV-VE; *AUC* Area under the receiver operation characteristics curve

### Diagnostic performance of FR

**Fig. 3:**
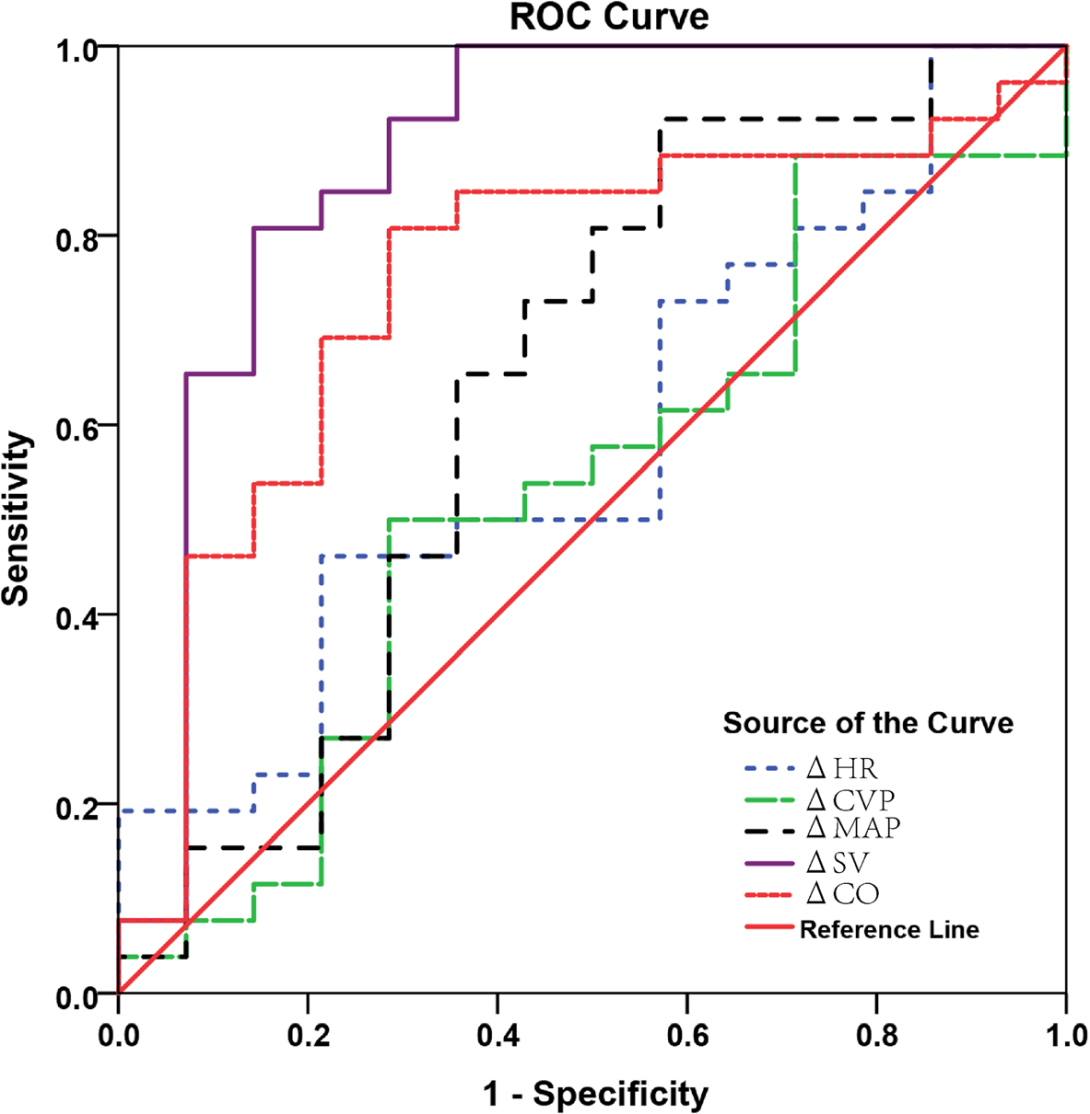
ROC curves comparing the ability of PLRinduced changes to discriminate responders from non-responders against VE. PLR: Passive leg raising; ROC: Receiveroperating characteristic; VE: Volume expansion

The optimal ΔSV-PLR threshold for predicting FR was 13 %, and its sensitivity and specificity were 81.8 and 86.3 %, respectively. The positive and negative predictive cut-offs were 85.0 and 82.0 %, respectively. The optimal threshold values and associated sensitivities and specificities are presented in Table 3; Fig. 3.

## Discussion

Prediction of FR is critical in fluid therapy after cardiac surgery, especially in children. Significant changes in ventricular systolic and diastolic functions after cardiac surgery often lead to difficulties in predicting FR [15, 16]. Here, we demonstrated that PLR related SV changes (ΔSV-PLR) as evaluated by bedside ultrasound could effectively predict FR in intubated children undergoing congenital heart surgery procedure. Our data showed that the optimal ΔSV-PLR threshold for predicting FR was 13 %.

PLR is a simple, noninvasive and repeatable method to change the cardiac preload [8, 9, 17]. Studies have shown that hemodynamic changes such as SV and aortic blood flow can be observed after 30 s of the elevation of lower limbs [18]. Our results showed that PLR can cause significant changes in hemodynamic parameters such as MAP, CVP, CO and SV (from base 1 to base 2), and all these parameters can also be restored to baseline level when the body position is returned to the Base 1 position (base 3 vs. base 1). This preliminary observation indicated that PLR could be used as a reversible fluid challenge.

In recent years, bedside ultrasonography has been considered as a noninvasive, real-time, convenient, low-cost, and repeatable tool for monitoring hemodynamics [10, 11, 19–21]. Previous studies have confirmed that echocardiography was highly correlated with PICCO in CO and SV, and PLR combined with non-invasive ultrasound has significant advantages in evaluating FR, which can be determined by monitoring SV and aortic blood flow [22–24]. The use of ΔSV-PLR to predict FR is based on the beneficial effects of cardiac preload on left ventricle function [25], which is not affected by changes in intrathoracic pressure, myocardial compliance, mechanical ventilation, or drug use. The best cut-off value for ΔSV in predicting FR fluctuates from 7 to 20 %, indicating a large variation among different studies [22–24]. Our study demonstrated that an increase of more than 13 % in the ΔSV-PLR can predict the FR in children after cardiac surgery, with a sensitivity of 81 %, a specificity of 86 %, and the AUC of 0.879.

Whereas CVP, MAP, and HR were relatively easy to monitor, we did not observe any correlation with ΔSV-VE. Our data showed that ΔCO-PLR could predict FR, with an optimal threshold of 8 %, sensitivity of 81 % and specificity of 71 %. But the correlation between ΔCO-PLR and ΔSV-VE was not comparable to that of the ΔSV-PLR, which may be due to the influence of the HR on CO measurement. Consistent with the previous findings, our study found that ΔSV-PLR can better predict FR compared with the other PLR-Δs [26].

There are some limitations in this study. First, echocardiographic measurement errors may have occurred, even though the same expert obtained all echocardiographic data. Second, because of the different types of congenital heart diseases and the diverse changes in cardiac structure after operation, our results cannot be extrapolated to other types of congenital heart diseases and to children of all ages, because leg elevation in newborns or infants obviously does not have the same volume effect as leg elevation in older children. Third, based on previous studies, we defined a 10 % increase in SV with rapid fluid loading as FR-positive. Whether the 10 % cut off is the appropriate threshold needs further research. Another limitation is that it is also a small sample, non-blinded study, and the results may not be applicable to other centers. Finally, someone is a fluid “responder” does not mean that they need a fluid bolus. If a healthy normally hydrated person is given a fluid bolus, their stroke volume will also increase, however, that does not mean that they need fluid. This is probably why the increases in MAP overall are very modest in our study even in responders. Therefore, further investigations of high-quality are needed to confirm our findings.

## Conclusion

In conclusion, our study demonstrated that assessment of the SV changes by noninvasive ultrasound combined with PLR could be used to evaluate fluid responsiveness in children under mechanical ventilation after congenital cardiac surgery.

## Data Availability

We declare that all relevant data and materials are available from the corresponding author on reasonable request.

## Abbreviations

ASD: Atrial septal defect
AUC: Area under the ROC curve
CVP: Central venous pressure
CO: Cardiac output
FR: Fluid responsiveness
HR: Heart rate
MAP: Mean arterial pressure
PiCCO: Pulse index Continuous Cardiac Output
PLR: Passive leg raising
ROC: Receiver operating characteristic
SV: Stroke volume
TTE: Transthoracic Doppler echocardiography
TOF: Tetralogy of fallot
VTI: Velocity-time integral
VE: Volume expansion
VSD: Ventricular septum defect
